# Global synchronous increase in light-saturated and peak vegetation productivity

**DOI:** 10.1016/j.fmre.2024.09.001

**Published:** 2024-09-05

**Authors:** Kun Huang, Jianyang Xia

**Affiliations:** a Zhejiang Tiantong Forest Ecosystem National Observation and Research Station, Research Center for Global Change and Complex Ecosystems, School of Ecological and Environmental Sciences, East China Normal University, Shanghai 200241, China; b Institute of Eco-Chongming (IEC), East China Normal University, Shanghai 200241, China

**Keywords:** Gross primary productivity, Peak vegetation productivity, Light-saturated productivity, Carbon fluxes, Synchronous increase

## Abstract

Terrestrial gross primary productivity (GPP), which refers to the photosynthetic uptake of CO_2_ by leaves, increases with incident irradiance and plateaus when leaves become light-saturated (GPP_sat_). While evidence suggests enhanced peak vegetation productivity (GPP_max_) since the 1980s, the existence of a light-saturation constraint on this enhancement remains unclear. Here, we combine the FLUXNET network’s 1269 site-years measurements with a flux-based gridded dataset to construct the first observationally derived light-response curves for global vegetation productivity. Our results show a synchronous increase of GPP_max_ and GPP_sat_ during the study period. There is convergence to a common ratio between GPP_max_ and GPP_sat_, with averaged ratio values of about 80% across global biomes. In particular, GPP_max_ was less light-saturated in evergreen broadleaved forests, suggesting significant potential for further productivity. The atmospheric CO_2_ fertilization effect was the primary driver of the synchronous increase in GPP_sat_ and GPP_max_, followed by temperature. Future projections by CMIP6 models indicate a continuing increase in GPP_max_ through the end of the 21st century. These findings illuminate the crucial role that rising peak and light-saturated vegetation productivity will play in sequestering atmospheric CO_2_ under future climate change scenarios.

## Graphical abstract

**Figure:**
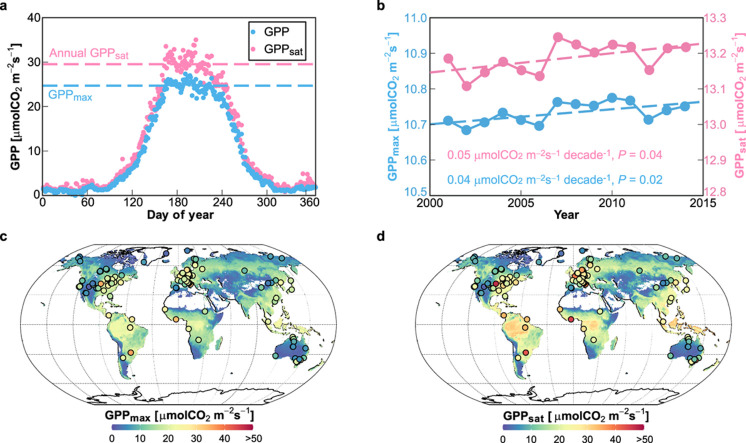

## 1 Introduction

The photosynthetic uptake of CO_2_ by all the plant leaves (gross primary productivity, GPP) is the largest CO_2_ flux in the global carbon (C) cycle, driving the capacity of land carbon sink to offset about one-third of the anthropogenic CO_2_ emissions [1,2]. The global vegetation photosynthesis is primarily dependent on the solar irradiance reaching the Earth’s surface [3]. Leaf-scale measurements show that the potential amount of C assimilated by vegetation photosynthesis tends to increase nonlinearly with incident solar irradiance until the leaves become light-saturated [4,5]. Analogous to light-saturated photosynthesis at the leaf level, ecosystem light-saturated GPP per ground area (GPP_sat_) is highly likely to be realized during the peak season because the intensity of incoming irradiance often peaks around the summer solstice across the north.

Vegetation photosynthesis usually reaches the peak (GPP_max_) during the summer over the whole growing season, and recent studies from global flux-tower measurements [6], satellite observations [7,8], and ecosystem modeling [7] show that atmospheric CO_2_ fertilizations have greatly increased GPP_max_ since the 1980s. Plot-level experiments identified GPP_sat_ as the upper layer well constraining seasonal trajectory of daily GPP values [[9], [10], [11]]. These multiple lines of evidence imply a potential light-saturated constraint on such an enhancement of the peak vegetation productivity over the global terrestrial ecosystems. Therefore, quantifying the global distribution of GPP_sat_, verifying the existence of light-saturated constraints on peak vegetation productivity in real-world ecosystems, and understanding the global relationships between GPP_sat_, light-saturated constraints on GPP_max_, and temporal enhancement of GPP_max_ are critical to understand and project temporal variations of annual productivity and land C sink [7,[12], [13], [14], [15]].

Using the well-documented light response curves (LRCs) to fit the half-hourly GPP time series from 177 eddy covariance towers of FLUXNET network [16,17] and FLUXCOM GPP data [18], we mapped global distributions of GPP_sat_ and GPP_max_. And then we investigated the temporal changes of global GPP_sat_ and GPP_max_. In this study, we present and test the following hypotheses: (1) widespread light-saturated constraints on GPP_max_ across global biomes, (2) global GPP_sat_ and GPP_max_ increase synchronously during the study period. Finally, we attributed the interannual variations in GPP_max_ and GPP_sat_ to the abiotic and biotic factors.

## 2 Materials and methods

### 2.1 Derivation of annual GPP_max_ and GPP_sat_ from flux tower measurements

We used eddy covariance (EC) network of CO_2_ exchanges between ecosystems and atmosphere provided by the FLUXNET 2015 Tier 1 dataset [19], and this standard dataset provides half-hourly and hourly ecosystem CO_2_ flux exchanges and meteorological recordings (downloaded from https://fluxnet.org/data/fluxnet2015-dataset/). 177 sites were selected according to the availability of EC flux measurements for at least 2 years. The global sites used cover a wide range of plant function types (Fig. S1), including croplands, grasslands, shrublands, savannas, evergreen and deciduous forests. The final number of sites selected was 177 (1269 site-years). We used half-hourly or hourly gross primary productivity derived from the nighttime flux partitioning method (GPP_NT_VUT_50, µmolCO_2_ *m*^−2^ *s*^−1^) and incoming photosynthetically active radiation (PAR, W *m*^−2^) to estimate the seasonal trajectory of GPP_sat_ for each selected site. As PAR measurements were not always available at the selected FLUXNET sites, we estimated PAR by multiplying incoming shortwave radiation (SW_IN_F, W *m*^−2^) by 0.45 [20].

We fit a non-rectangular hyperbolic light response curve (NHLRC) to half-hourly or hourly GPP and solar radiation data as follows [21]:

(1) $$\text{GPP}=\frac{1}{2\theta }\left(\alpha Q+\beta -\sqrt{{\left(\alpha Q+\beta \right)}^{2}-4\alpha \beta \theta Q}\right)$$

where α is the initial slope of the light response curve, θ is the curvature parameter (ranging from 0 to 1), β is the plateau of the light response curve, GPP is the half-hourly or hourly GPP values, and Q is the incoming PAR to drive the model.

For each year, using PAR (converted from incoming solar radiation) as driving radiation, the parameters of the NHLRC were estimated. We calculated the GPP at a PAR threshold of 2000 µmol *m*^−2^ *s*^−1^ as saturating light (GPP_sat_) [12]. The LRC was fit to 5 days of data selected with a moving window approach. The estimated parameters and the GPP_sat_ values were assigned to the day in the middle of the 5-day window (Fig. S2). A time series of daily values of GPP_sat_ was then derived for each year.

Following the previous study [20], we concluded that the 90th percentile of the derived GPP_sat_ parameters of NHLRCs was selected as an appropriate approach to represent ecosystem photosynthetic capacity. The 90th percentile of the time series of estimated daily GPP_sat_ estimates was then extracted as the corresponding annual GPP_sat_ for each site-year. The 90th of half-hourly or hourly daytime GPP (incoming shortwave radiation greater than 200 W *m*^−2^) was calculated as annual maximum realized GPP (GPP_max_) of each site-year. Finally, we calculated the annual ratio of GPP_max_ to GPP_sat_ for each site-year [22].

### 2.2 Derivation of annual GPP_max_ and GPP_sat_ from FLUXCOM dataset

We utilized a half-hourly flux-based gridded GPP product during 2001–2014 (downloaded from https://doi.org/10.17871/BACI.224), provided by the Max Plank Institute for Biogeochemistry (MPI-BGC) FLUXCOM initiative with a spatial resolution of 0.5° × 0.5° This dataset allows us to analyze the diurnal cycling of ecosystem photosynthesis globally [19]. We downloaded hourly PAR recordings from the second Modern-Era Retrospective analysis for Research and Applications (MERRA-2) at a spatial resolution of 0.625° (longitude) by 0.5° (latitude) during 2001–2014. We first aggregated the hourly PAR to half-hourly interval temporal resolution, and then variables were resampled to the spatial resolution of 0.5° × 0.5° using the bilinear interpolation method.

For the annual cycle of each grid cell, we fit the NHLRC to half-hourly GPP and PAR data with the same fitting strategy as for in situ EC flux measurements. Then we derived the daily values of fitted GPP_sat_ parameter of NHLRCs for a specific year. The 90th percentile of the daily values of estimated GPP and GPP_sat_ was then extracted as the pixel-level annual GPP_max_ and GPP_sat_.

### 2.3 Forcing datasets

Forcing datasets were utilized to investigate the relative importance of environmental and vegetation factors in driving annual changes in GPP_max_ and GPP_sat_, including incident photosynthetic active radiation (PAR; W *m*^−2^), mean annual temperature (MAT; °C), mean annual temperature (MAT; °C), aridity index, mean annual vapor pressure deficit (VPD; kPa), soil water content (SWC; m^3^ *m*^−3^), atmospheric CO_2_ concentration (CO_2_; µmol mol^−1^), maximum leaf area index (LAI_max_; m^2^ *m*^−2^) and terrestrial alive vegetation biomass (Biomass; Mg C ha^−1^). The atmospheric CO_2_ concentration data were downloaded from CarbonTracker CT2019B [23]. The annual surface air temperature data with a spatial resolution of 0.5° × 0.5° were obtained from meteorological data stored at the Climate Research Unit, University of East Anglia (CRU TS 4.02). The incoming PAR was derived from a 50-km gridded MERRA-2 dataset. The annual SWC data were obtained from GLEAM (www.gleam.eu) with a spatial resolution of 0.5° × 0.5° The monthly VPD data were obtained from TerraClimate with a spatial resolution of 1/24° Annual LAI_max_ was retrieved from MOD15A2H version 6 dataset. The annual terrestrial alive biomass was obtained with a spatial resolution of 0.1° × 0.1° [24]. All the monthly gridded datasets were averaged into annual values, and then spatially aggregated to a resolution of 0.5° × 0.5°to match the derived annual GPP_max_ and GPP_sat_.

### 2.4 Statistical analyses and relative importance calculations

All the statistical analyses and relative importance calculations were conducted using *R* (http://www.r-project.org/). The temporal trends were estimated by the Theil–Sen slope estimator (referred to here as Sen’s slope) [25]. The statistical differences between GPP_max_ and GPP_sat_ for each flux site or grid cell were tested using the paired *T*-test.

We conducted a random forest regression analysis to identify the most important predictors of annual changes in GPP_max_ and GPP_sat_. The random forest analysis could account for interactions and nonlinear relationships between predictors, and could deal with the multicollinearity problems in multivariate regression [26]. The fit for each tree is determined by randomly selecting cases. The importance of each predictor variable is determined by the percentage increase in the mean square error (%IncMSE) between observations and predictions, and the decrease is averaged over all the trees to produce the final estimation for importance. Greater values of%IncMSE denote higher variable importance. In this study, the importance measure was calculated for each tree and averaged over the forest (1000 trees). These variable importance analyses were conducted using the randomForest package in *R*.

### 2.5 Future projected GPP_max_ by Earth system models

We used monthly outputs of GPP from 10 Earth system models (ESMs) participating in the 6th phase Coupled Model Intercomparison Project (CMIP6) under the historical period (2001–2014) and shared socioeconomic pathway (SSPs) 5–8.5 scenario (2015–2100), covering the time period from 2001 to 2100 (Table S1). All ESM outputs can be downloaded from the Institute Pierre-Simon Laplace server (https://esgf-node.ipsl.upmc.fr/search/cmip6-ipsl/). Monthly GPP for the vegetated lands was bilinearly interpolated to 0.5°  ×  0.5° in longitude and latitude, and then aggregated to obtain the maximum value (GPP_max_) on a yearly time scale.

## 3 Results

### 3.1 Synchronous increase of light-saturated and peak vegetation productivity

**Fig. 1:**
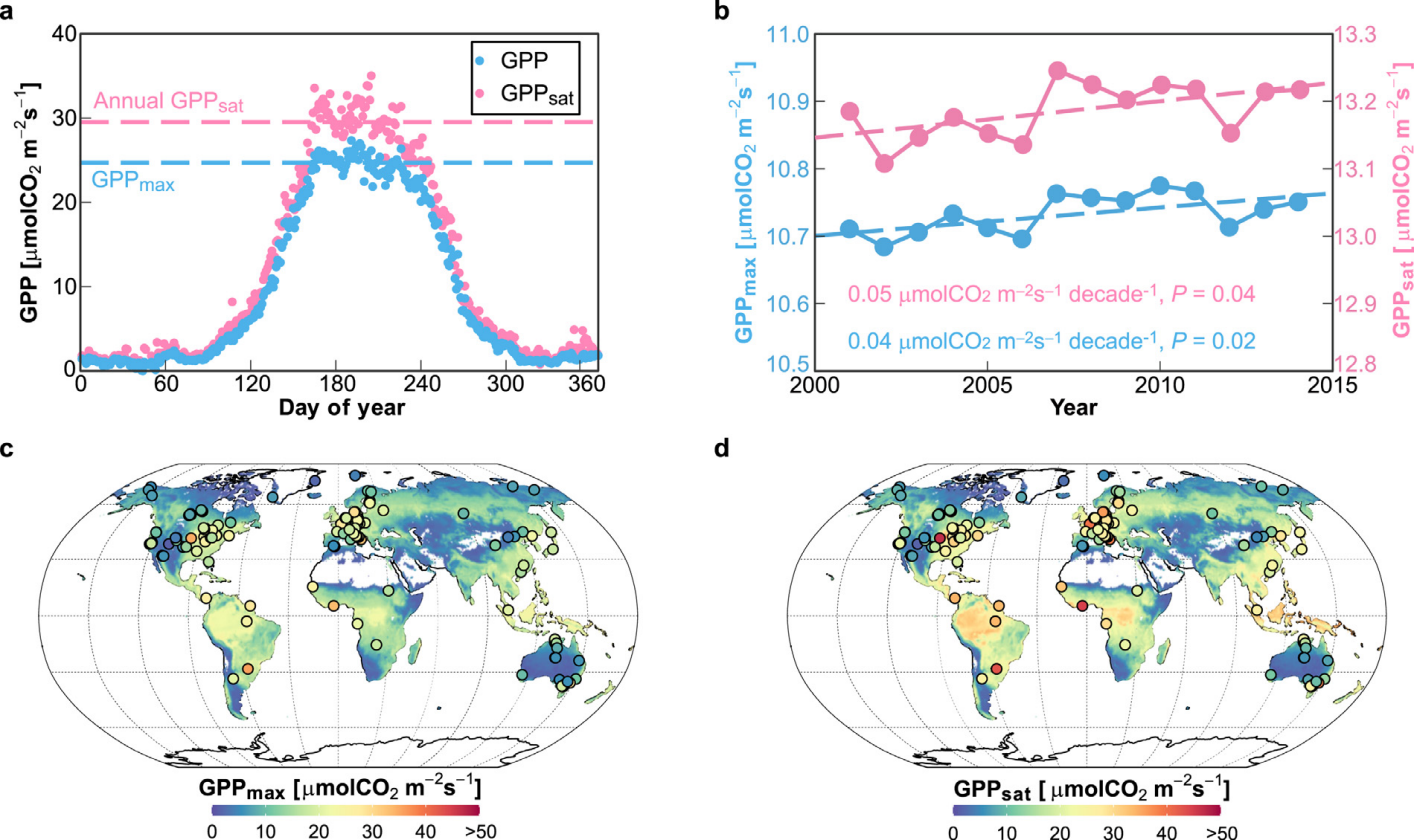
**Synchronous increase of peak seasonal GPP (GPP_max_) and light-saturated GPP (GPP_sat_) derived from global flux-tower sites and FLUXCOM dataset for half-hourly GPP.** (a) Seasonal trajectory of daily GPP and GPP_sat_ at a temperate forest site (CN—Cha; 42.4°N, 128.1°E). Annual GPP_sat_ is calculated from the half-hourly data by fitting the non-rectangular hyperbolic light response curve with a moving window of 5 days. Then the 90% percentiles of annual GPP and GPP_sat_ time series are then extracted as the GPP_max_ and GPP_sat_ for each site-year, respectively. (b) Annual time series of pixel-averaged GPP_max_ and GPP_sat_ derived from FLUXCOM GPP data. The solid lines with markers indicate GPP_max_ and annual GPP_sat_, and the dotted lines represent linear regression. (c) and (d), Spatial distributions of GPP_max_ (b) and annual GPP_sat_ (c). The circles on the map are colored according to the local value retrieved from each FLUXNET tower site.

We first derived seasonal trajectory of GPP and GPP_sat_ at 177 FLUXNET eddy covariance (EC) sites (Fig. 1a), and identified the GPP_max_ and annual GPP_sat_ for each flux site (Fig. 1c, d). FLUXNET GPP_max_ ranged from 1.54 µmolCO_2_ *m*^−2^ *s*^−1^ to 45.04 µmolCO_2_ *m*^−2^ *s*^−1^, and annual GPP_sat_ ranged from 1.77 µmolCO_2_ *m*^−2^ *s*^−1^ to 60.88 µmolCO_2_ *m*^−2^ *s*^−1^ (Fig. 1c, d). Flux tower-derived seasonal trajectory revealed that annual GPP_sat_ was greater than GPP_max_ for each plant function type (PFT) (Fig. S3). This field evidence suggested that GPP_sat_ could well act as one upper layer for apparent GPP and GPP_max_. The in-situ EC flux measurements robustly provided observational support to further verify the existence of possible light-saturated constraints on GPP_max_ across global ecosystems.

**Fig. 2:**
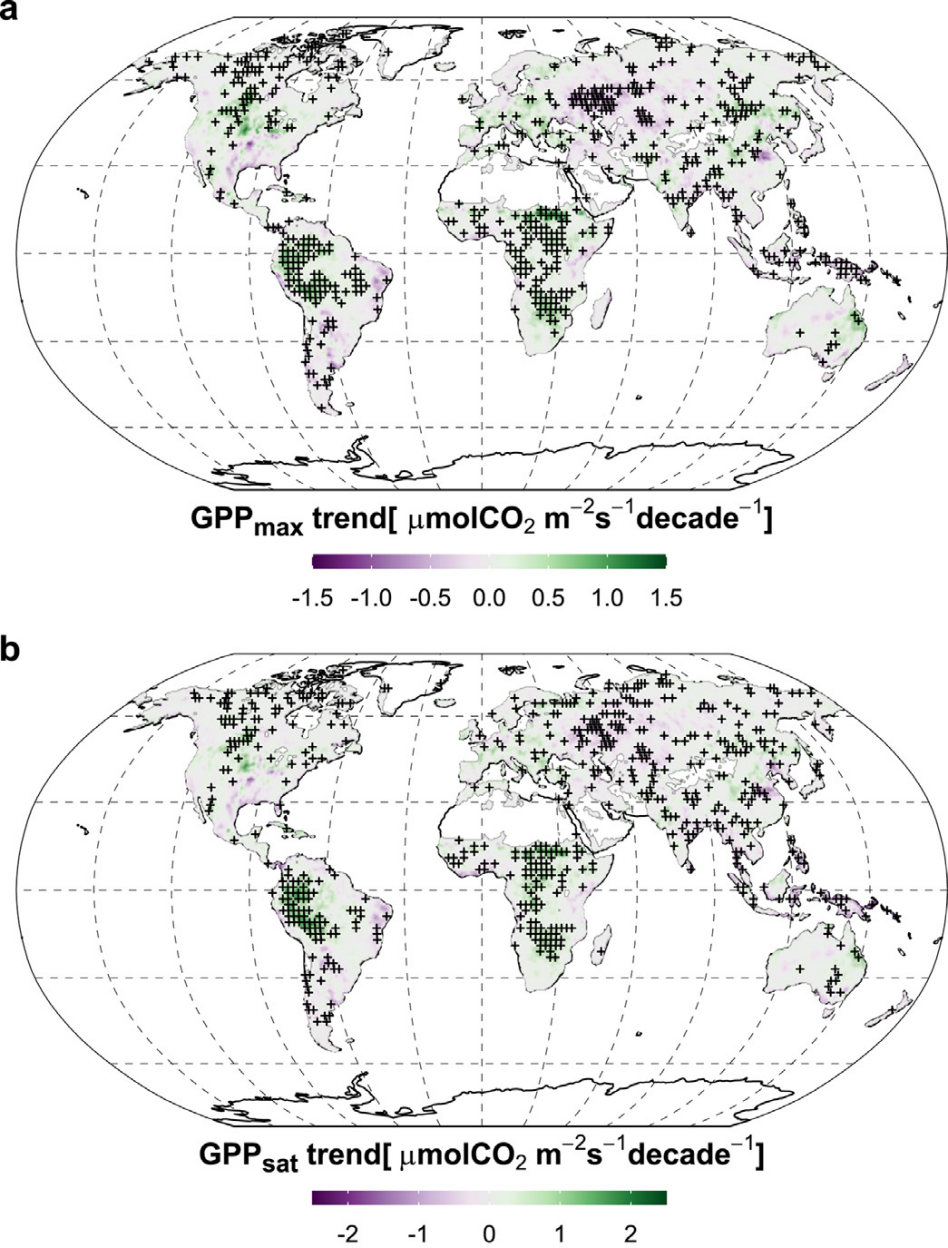
**Global patterns in trends of annual GPP_max_ (upper panel) and GPP_sat_ (bottom panel) over the period 2001–2014 using half-hourly FLUXCOM dataset**. The changing trends are calculated on a per-pixel basis (0.5° × 0.5°) over time and dotted if statistically significant (*P* < 0.1).

Since in situ flux towers do not have continuous spatial coverage, we also used FLUXCOM half-hourly GPP to derive the global distributions of GPP_sat_ (Fig. 1c, d). Temporally, GPP observations from FLUXCOM dataset suggested that GPP_max_ and annual GPP_sat_ significantly increased during 2001–2014, respectively (Fig. 1b). Global-averaged GPP_max_ increased with a rate of 0.04 µmolCO_2_ *m*^−2^ *s*^−1^ per decade (*P* = 0.02), and the annual GPP_sat_ increased with a relatively faster rate of 0.05 µmolCO_2_ *m*^−2^ *s*^−1^ per decade (*P* = 0.04). Spatially, global-averaged GPP_max_ and annual GPP_sat_ were 10.7 ± 6.23 µmolCO_2_ *m*^−2^ *s*^−1^ and 13.22 ± 8.16 µmolCO_2_ *m*^−2^ *s*^−1^, respectively (Fig. 1b, c). Spatially, increasing GPP_max_ and GPP_sat_ were also identified widespread over the globe (Fig. 2).

### 3.2 Convergence in light-saturated constraints to GPP_max_ across global biomes

**Fig. 3:**
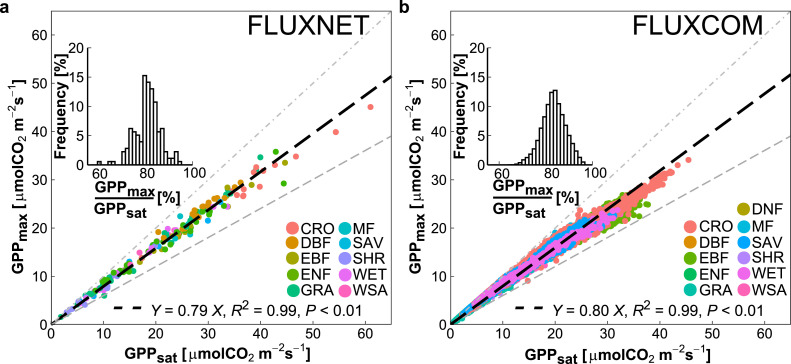
**Global relationships between GPP_max_ and annual GPP_sat_ from FLUXNET network and FLUXCOM dataset.** The relationship between GPP_max_ and GPP_sat_ is shown across (a) all FLUXNET sites and (b) all 0.5° × 0.5° vegetated pixels for each PFT. The two light gray dotted lines represent *Y* = *X* and *Y* = 0.6*X*, respectively. The black dotted lines are derived by linear regression. *Insets* show the relative frequency distributions of ratios (GPP_max_/GPP_sat_) from all FLUXNET site-years and FLUXCOM dataset, respectively.

To explore how the terrestrial vegetation achieved the global synchronous increase of GPP_max_ and GPP_sat_ during the study period, we assessed relationships between GPP_sat_ and GPP_max_ (ratio of GPP_max_ to annual GPP_sat_) across global PFTs (Fig. 3). Global-distributed flux towers and FLUXCOM dataset consistently showed that the linear relationships between GPP_sat_ and GPP_max_ converged across a broad range of PFTs and environmental types (Figs. 3 and S4). The GPP_sat_-GPP_max_ slope fitted to the global-distributed flux measurements (0.79) was similar to the slope fitted to the FLUXCOM vegetated grid cells (0.8) over the globe, with the same explanatory power (*R*^2^ = 0.99). The most frequent ratio value was 80%, with 90% of the ratio values falling with a range from 72% to 88% based on an analysis of FLUXNET 177 sites (Fig. 3a, *Inset*). Similarly, the global FLUXCOM dataset showed convergent ratios across vegetated grid cells (Fig. 3b), with the most frequent ratio value of 80% and a range from 75% to 91% (Fig. 3b, *Inset*). Overall, the observationally convergent relationships between GPP_sat_ and GPP_max_ validated the ecosystem-level existence of light-saturated constraints on peak vegetation productivity.

**Fig. 4:**
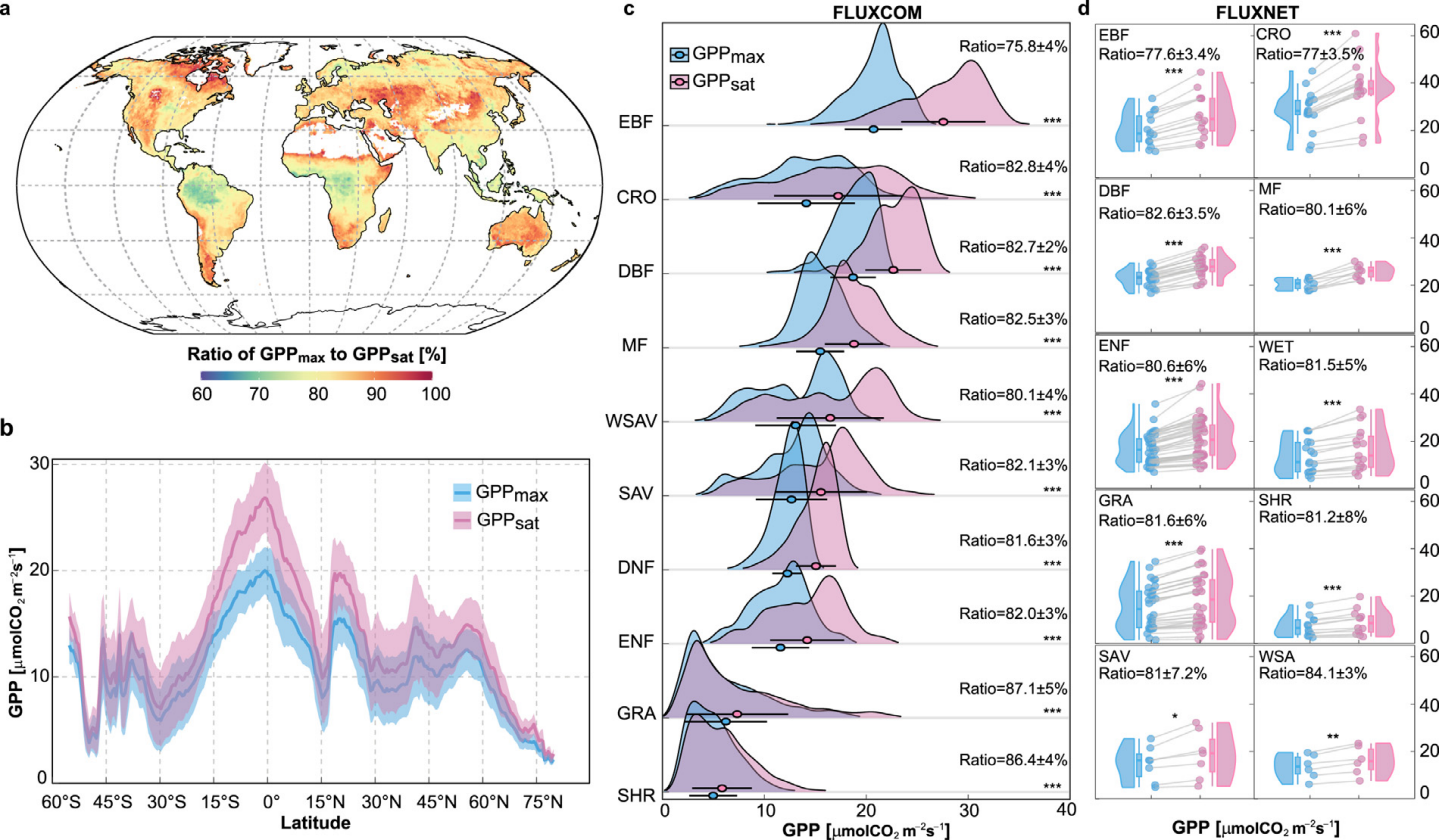
**Global relationships between GPP_max_ and annual GPP_sat_ from FLUXCOM (a, b, c) and FLUXNET network (d) across global biomes.** (a) Global distribution of GPP_max_/GPP_sat_ ratios. (b) Latitudinal distributions of GPP_max_ and annual GPP_sat_ from FLUXCOM dataset. Values are first calculated pixel by pixel and then averaged by latitude. The solid line and shaded area indicate the mean and half s.d. of GPP summarized by latitude, respectively. (c) Biome-level distribution of pixel-aggregated GPP_max_ and annual GPP_sat_ from FLUXCOM GPP. (d) Biome-level distribution of GPP_max_ and annual GPP_sat_ from 177 FLUXNET sites. For boxplots, the cross indicates the mean, the center line indicates the median, the box indicates the upper and lower quartiles and the whiskers indicate the 5th and 95th percentiles of the data. The number of sites distributed in each panel. Differences between the GPP_sat_ and GPP_max_ for each biome were significant using a paired *t*-test. *P  <  0.05; **P  <  0.01; ***P  <  0.001.

To further identify the possible hotspots of space for enhanced GPP_max_, we globally mapped the ratios of GPP_max_ to annual GPP_sat_ derived from FLUXCOM GPP data (Fig. 4). Across the latitudes, due to the widely-distributed evergreen broadleaf forests (EBF) in those regions (Fig. 4b, c), relatively lower ratio values (75.8%) were found in the tropical and subtropical climate zones (Fig. 4b, c). Then the ratio values increased to 87% (lower GPP_max_ and GPP_sat_) and gradually approached 100% toward a latitude of 20°S southward (Fig. 4b). Higher ratio-values with a range from 86.5% to 87.1% were found at the semi-arid ecosystems, including grassland (GRA) and shrubland (SHR) (Fig. 4c).

FLUXNET-derived ratios of GPP_max_ to annual GPP_sat_ were convergent at the ratio-value of 80% across the 10 PFTs (Fig. 3d). In addition, slightly lower ratio-values were identified at EBF sites (77.6% ± 3.4%) and cropland sites (77% ± 3.5%), respectively (Fig. 4d). Overall, observations from FLUXCOM and FLUXNET showed that GPP_max_ from dense canopies (e.g., EBF) was less aligned with GPP_sat_, implying that EBFs may have large space for enhancement on GPP_max_ in the future.

### 3.3 Attributions of the annual changes in GPP_max_ and GPP_sat_

**Fig. 5:**
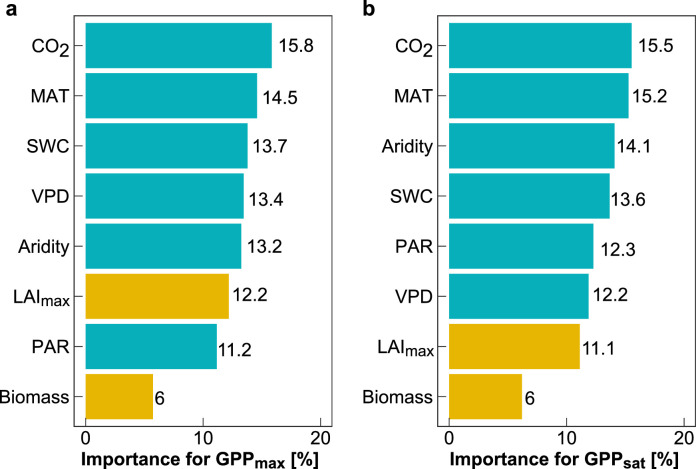
**Attributions to annual changes of GPP_max_ and GPP_sat_ during 2001–2014.** Annual changes are calculated as the difference between the time period (2012–2014) and time period (2001–2003). Relative importance for GPP_max_ (a) and GPP_sat_ (b). Orange bars represent vegetation structural variables; light blue bars represent climate variables.

We calculated annual changes in vegetation and environmental variables for each grid cell between the two time periods, and further explored the relative importance of biotic and abiotic factors to annual changes of GPP_sat_ and GPP_max_ using the random forests (Fig. 5). Over 70% of the annual changes in GPP_max_ and GPP_sat_ were explained by the environmental factors. Annual changes of GPP_max_ were dominated by CO_2_ fertilization (15.8%), followed by air temperature (14.5%), and lastly by water availability (SWC, VPD, and Aridity). Similarly, annual variations in GPP_sat_ were dominated by CO_2_ fertilization (15.5%), followed by air temperature (14.5%), and lastly by water availability (Aridity, SWC, and VPD) and incident solar irradiance (12.3%). Overall, the attributed results showed that the CO_2_ fertilization largely contributed to the synchronous increase of GPP_max_ and GPP_sat_ over the globe.

## 4 Discussion and conclusion

Using the two observational datasets, our global estimates of GPP_sat_ and its ratio value to GPP_max_ are to our knowledge the first such spatially-resolved investigation. We successfully derived the GPP_sat_ as one upper layer for GPP_max_ across the globe (Fig. 1). Given that enhanced GPP_max_ has been observed since the 1980s, our study provides a novel approach to investigate the light-saturated constraint on the GPP_max_ enhancement. Interestingly, our analysis reveals a synchronous increase of global GPP_sat_ and GPP_max_, and we also identify the global convergence in ratios of GPP_max_ to GPP_sat_.

First, annual changes of GPP_max_ and GPP_sat_ are mainly determined by atmospheric CO_2_ fertilization, climatic factors, and vegetation’s structure properties (Fig. 5). GPP could be estimated by the light use efficiency (LUE) framework, which considers plants as biological solar collectors that convert sunlight absorbed by leaves into chemical energy [27]. Then GPP_max_ could be jointly controlled by canopy structure and LUE [14], which are influenced by environmental changes and vegetation’s biochemical properties [2,[28], [29], [30]]. Seasonal trajectory of environmental conditions could influence GPP_max_ by supplying energy (solar radiation), canopy structure development (LAI), and regulating LUE [31]. Field measurements have reported that plant photosynthesis increases with incoming sunlight, and GPP gets saturated (GPP_sat_) until all the leaves become light-saturated [9,32]. LAI_max_ mediated the structural capacity of vegetation to convert sunlight to photosynthate at the ecosystem scale. In terms of converting to chemical energy, vegetation LUE is a key physiological representation of the photosynthetic processes [[33], [34], [35], [36], [37], [38]]. Our study emphasizes the vegetation structural and physiological controls on peak vegetation productivity.

Second, the global convergence in the ratio of GPP_max_ to GPP_sat_ robustly verifies the existence of light-saturated constraints on GPP_max_, probably leading to the synchronous increase of GPP_sat_ and GPP_max_. The widespread existence of light-saturated constraints on GPP_max_ provides observational support for the global synchronous of GPP_sat_ and GPP_max_ (Fig. 3). The global light-saturated constraint might imply that the space for further GPP_max_ enhancement may be widely limited by biophysical and ecological factors (Fig. 5). This could be partly due to the seasonal synergy between the maximum canopy structure and highest environmental resources availability/use efficiency. Satellite observations have reported the timing asynchrony between the maximum absorbed PAR by canopy leaves (scaled with LAI_max_) and peak photosynthesis due to the environmental constraints [39], and seasonality of light-use efficiency influenced the decoupling of maximum canopy structure and peak vegetation productivity [40]. Based on satellite and flux towers observations, the widespread mismatch between maximum canopy structure and optimal resource availability (e.g. light, nutrient and water) was likely to cause the suboptimal GPP_max_ over the northern ecosystems [8,31]. In particular, the slightly less light-saturated GPP_max_ in EBF was likely due to the dominant role of light limitations on vegetation productivity in those regions [41]. Then the complex canopy structure of EBFs (mostly mature forests) often hampers the sunlight penetrating into the understory plants, which also contributes to the vegetation productivity at the whole ecosystem level [42]. Overall, the space for GPP_max_ enhancement is largely determined by canopy structural properties and environmental conditions.

**Fig. 6:**
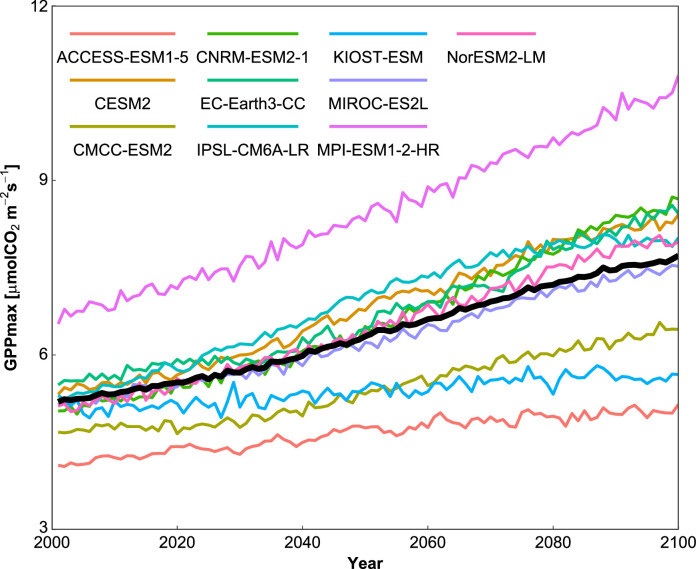
**Future projections of increasing GPP_max_ using the 10 CMIP6 ESMs for 2001–2100 under the SSP5–8.5 scenario.** The black line represents the mean values of the 10 models.

Third, the global synchronous increase of GPP_sat_ and GPP_max_ has important implications for future projections of the plant’s CO_2_ uptake and global C cycle. Previously, we have reported that atmospheric fertilization effects led to the increasing GPP_max_ over the three past decades [7]. Here, using the well-documented LRCs theory of vegetation physiology, this study showed global GPP_sat_ increased slightly faster than GPP_max_ during 2001–2014 (Fig. 1b), and the convergent ratios between GPP_sat_ and GPP_max_ across the globe could well explain this observed synchronous increase. The global mapping of ratios implies that there are room for further enhancement on GPP_max_. The future increasing GPP_max_ projected by multiple CMIP6 earth system models further confirmed the continuously rising peak vegetation productivity, highlighting the positive contributions of GPP_max_ to the potential for plant’s CO_2_ uptake under the future warming scenario (Fig. 6).

Uncertainties are acknowledged here. Temporally, our approach of using the FLUXCOM dataset may underestimate the temporal trend of peak photosynthesis and its light saturation. Although the LRC theory is well-documented, it relies on the diurnal GPP measurements. There is no long-term half-hourly GPP at the global scale, and the current FLUXCOM dataset fails to account for CO_2_ fertilization [43]. Future satellite observations (for example, solar-induced chlorophyll fluorescence signals) for diurnal patterns of vegetation photosynthesis with global coverage will allow us to detect the long-term GPP_max_ and GPP_sat_ [44]. Yet, the convergent relationships between GPP_sat_ and GPP_max_ across global PFTs provide observational support for the synchronous increase of GPP_sat_ and GPP_max_ [43].

Peak productivity represents the most principal axis of terrestrial ecosystem functions [13], and increasing the peak vegetation productivity is the most obvious way to remove CO_2_ from the atmosphere [45]. Also, light-saturated vegetation productivity increased faster than vegetation productivity, implying that light-saturated vegetation productivity will act as a potential upper layer for peak productivity. Our study provided a perspective on how the plants will achieve peak productivity in the long run. Global estimates of light-saturated and peak vegetation productivity also provided a critical link for understanding the potential for ecosystems to mine CO_2_ from the atmosphere [46].

The major contribution of our study is to present a clear and spatially-resolved picture of the synchronous increase of light-saturated and peak vegetation productivity. Our annual mapping of GPP_max_ and GPP_sat_ offers observational support to quantifying the light-saturated constraint for peak vegetation productivity. This could aid in quantifying the most important representation of terrestrial ecosystem functions and its global drivers, and can also help reduce the uncertainty of ecosystem carbon stock projections under the prevailing land greening [47].

## Data availability statement

The global half-hourly GPP products are available at https://doi.org/10.17871/BACI.224. The eddy covariance measurements provided by the FLUXNET2015 Tier1 dataset are from https://fluxnet.org/data/fluxnet2015-dataset/. The global surface air temperature and precipitation datasets are from https://crudata.uea.ac.uk/cru/data/hrg/. The MERRA-2 incoming PAR is from https://goldsmr4.gesdisc.eosdis.nasa.gov/data/MERRA2/. The GLEAM root-zone SWC dataset is from https://www.gleam.eu/. The VPD dataset from TerraClimate is available at https://www.climatologylab.org/terraclimate.html. The gridded aridity dataset is from https://cgiarcsi.community/data/global-aridity-and-pet-database/. MODIS LAI dataset is from https://lpdaac.usgs.gov/products/mod15a2hv006/. The global gridded CO2 concentration was downloaded from CarbonTracker CT2019B at http://carbontracker.noaa.gov. The annual terrestrial alive biomass is from https://zenodo.org/records/4,161,694.

## CRediT authorship contribution statement

**Kun Huang:** Conceptualization. **Jianyang Xia:** Conceptualization.

## Declaration of competing interest

The authors declare that they have no conflicts of interest in this work.

## Appendix Supplementary materials

Image, application 1
