# Supplementary material for: Global synchronous increase in light-saturated and peak vegetation productivity
Source: Fundam Res. 2024 Sep 5;6(3):1545–51. doi: 10.1016/j.fmre.2024.09.001 (PMC13247453; doi:10.1016/j.fmre.2024.09.001)
Supplement: Supplementary file 1 [file mmc1.docx]

Supplementary information

**Global synchronous increase in light-saturated and peak vegetation productivity**

Kun Huang^1,2,3^, Jianyang Xia^1,2^

^1^ Zhejiang Tiantong Forest Ecosystem National Observation and Research Station, School of Ecological and Environmental Sciences, East China Normal University, Shanghai 200241, China;

^2^ Research Center for Global Change and Complex Ecosystems, East China Normal University, Shanghai 200241, China;

^3^ Institute of Eco-Chongming (IEC), East China Normal University, Shanghai 200241, China.

* Corresponding author: [jyxia@des.ecnu.edu.cn](mailto:jyxia@des.ecnu.edu.cn) (Jianyang Xia)

This PDF file include:

3 Figure

1 Tables

10 References

**Supplementary Figure 1. Global distribution of FLUXNET sites used in this study across the PFTs.** Each PFT: CRO (Croplands); DBF (deciduous broadleaved forest); EBF (evergreen broadleaved forest); ENF (evergreen needleleaf forest); GRA (grasslands); MF (mixed forest); SAV (savannah); SHR (shrublands); WET (wetlands); and WSA (woody savannah).


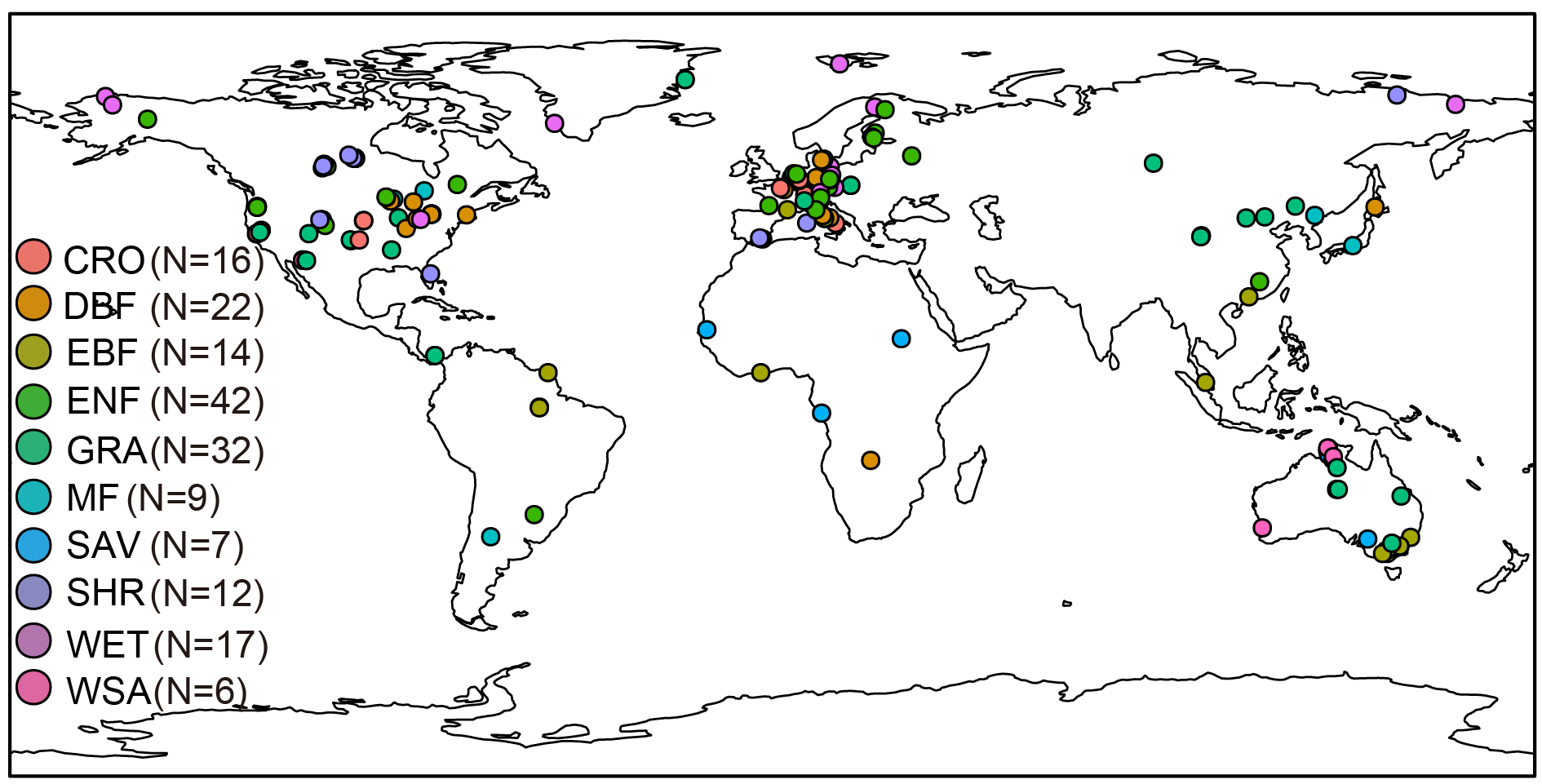


**Supplementary Figure 2. A case showing the use of light response curve to detect the ecosystem-scale daily value of light-saturated GPP (GPP_sat_) at a temperate forest site** (CN-Cha; 42.4°N, 128.1°E)**.** Black dots indicate the observed half-hourly GPP and the corresponding PAR from the Changbaishan flux tower during the specific time interval (DOY170-DOY176) for the year of 2005. DOY: Day of year


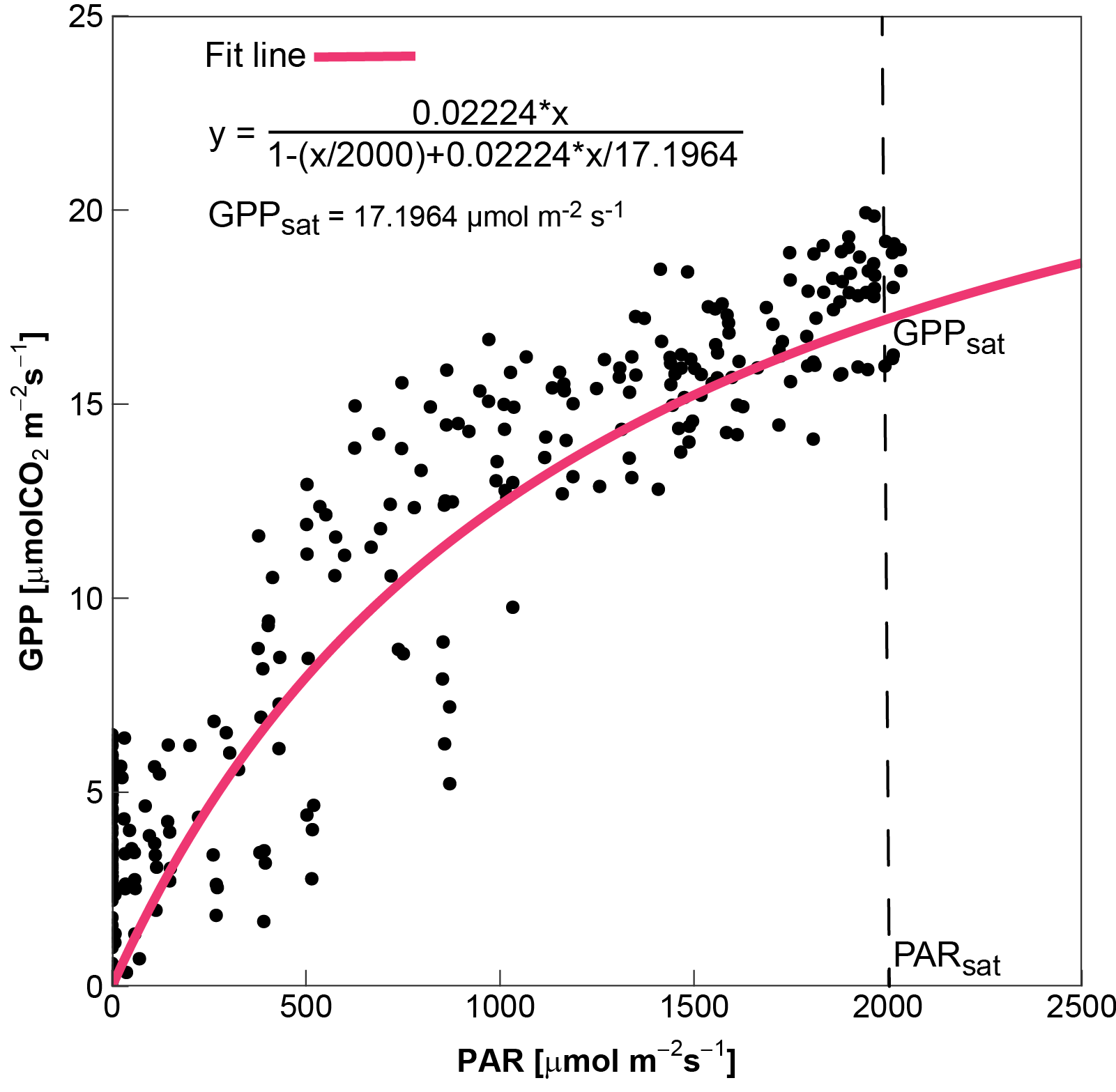


**Supplementary Figure 3.** Seasonal trajectory of light-saturated GPP (GPP_sat_) and apparent GPP for each plant functional type (PFT) at 177 FLUXNET eddy covariance sites. PFTs: CRO (Croplands); DBF (deciduous broadleaved forest); EBF (evergreen broadleaved forest); ENF (evergreen needleleaf forest); GRA (grasslands); MF (mixed forest); SAV (savannah); SHR (shrublands); WET (wetlands); and WSA (woody savannah).

**
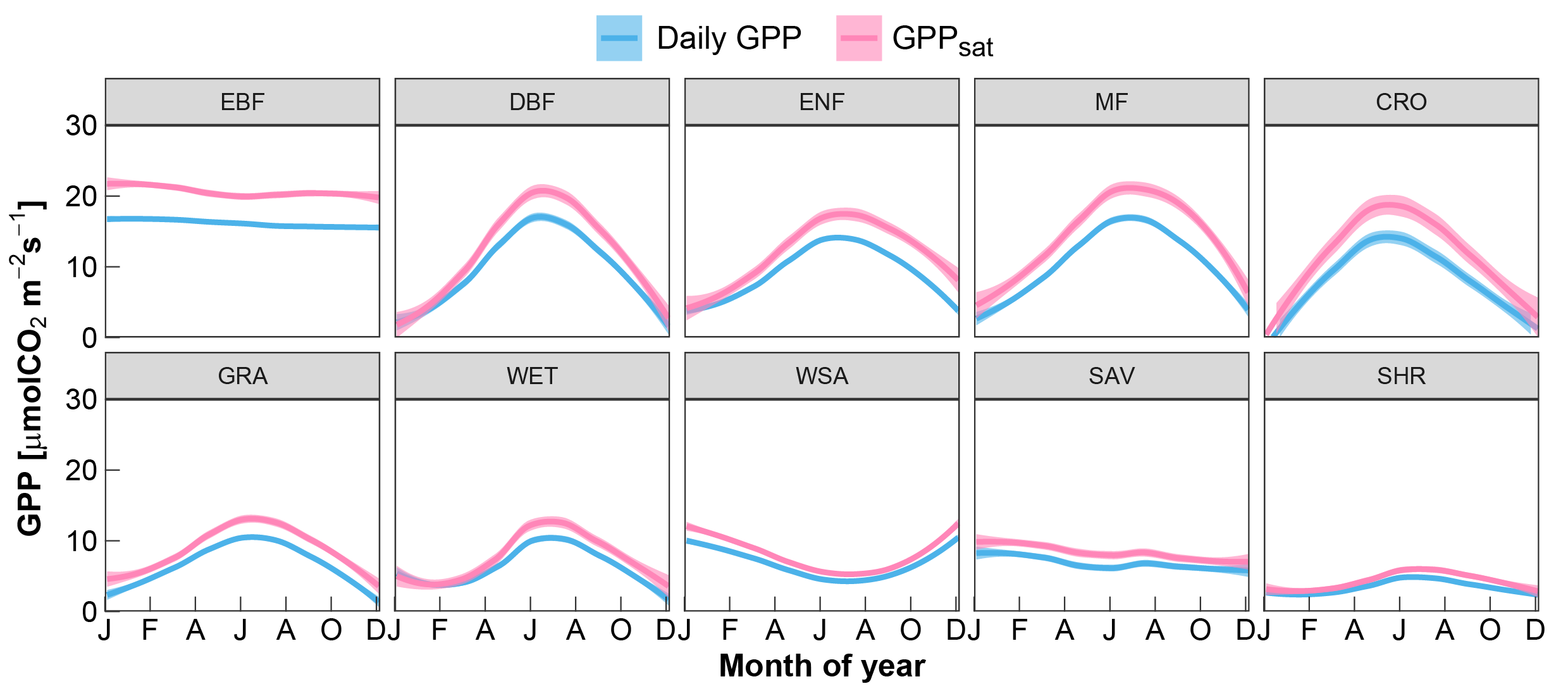
**

**Supplementary Figure 4. The MODIS land cover type at the resolution of 0.5°×0.5°.** Biome maps constructed from the MODIS/TERRA-AQUA land cover 7 product (MCD12C1) Collection 5.1. Plant functional types: ENF (evergreen needleleaf forest), EBF (evergreen broadleaf forest), DNF (deciduous needleleaf forest), DBF (deciduous broadleaf forest), MF (mixed forest), SHR (open shrublands and closed shrubland), SAV (savanna and woody savannas), GRA (grassland), WET (wetland), CRO&URB (cropland and urban), ICE (ice and snow), and BAR (barren and sparsely vegetated).


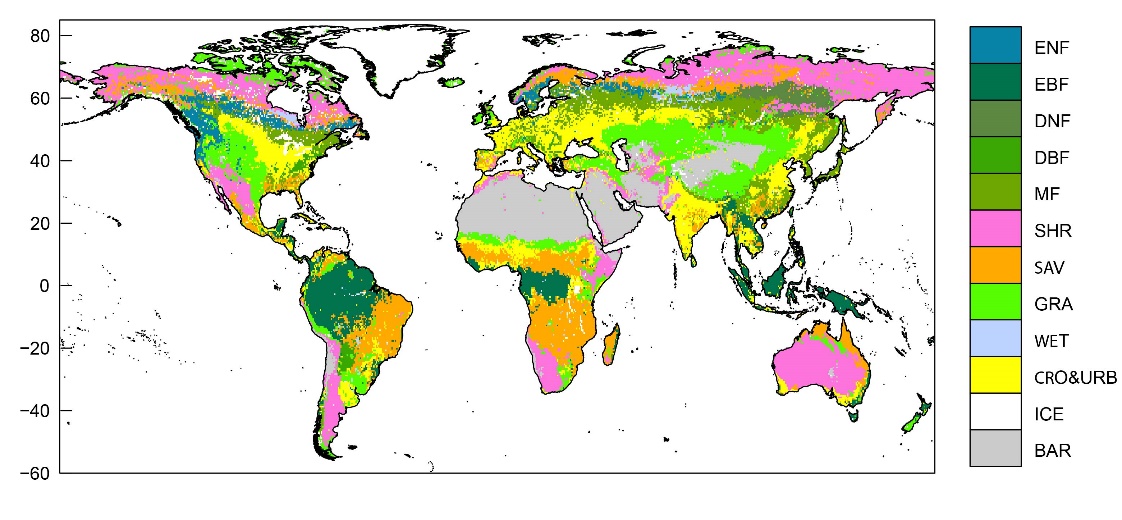


**Supplementary Table 1.** Ten CMIP6 earth system models used in this study.

| **Model** | **Data period** | **Variable** | **Time frequency** | **Reference** |
| --- | --- | --- | --- | --- |
| ACCESS-ESM1-5 | 2001-2100 | GPP | Monthly | Law at al. 2017 (ref^1^) |
| CESM2 | 2001-2100 | GPP | Monthly | Lawrence et al. 2019 (ref^2^) |
| CMCC-ESM2 | 2001-2100 | GPP | Monthly | Lovato et al. 2022 (ref^3^) |
| CNRM-ESM2-1 | 2001-2100 | GPP | Monthly | Séférian et al. 2019 (ref^4^) |
| EC-Earth3-CC | 2001-2100 | GPP | Monthly | Döscher et al. 2022 (ref^5^) |
| IPSL-CM6A-LR | 2001-2100 | GPP | Monthly | Vuichard et al. 2019 (ref^6^) |
| KIOST-ESM | 2001-2100 | GPP | Monthly | Pak et al. 2021 (ref^7^) |
| MIROC-ES2L | 2001-2100 | GPP | Monthly | Hajima et al. 2019 (ref^8^) |
| MPI-ESM1-2-HR | 2001-2100 | GPP | Monthly | Mauritsen et al. 2019 (ref^9^) |
| NorESM2-LM | 2001-2100 | GPP | Monthly | Seland et al. 2020 (ref^10^) |

**Supplementary References**

1. R. M. Law, et al. The carbon cycle in the Australian Community Climate and Earth System Simulator (ACCESS-ESM1)—Part 1: Model description and pre-industrial simulation. Geosci. Model. Dev. 10 (2017) 2567-2590.
2. D. M. Lawrence, et al. The Community Land Model version 5: Description of new features, benchmarking, and impact of forcing uncertainty. J. Adv. Model. Earth Syst. 1 (2019) 245-4287.
3. T. ovato, et al. CMIP6 simulations with the CMCC Earth System Model (CMCC-ESM2). J. Adv. Model. Earth Syst. 14 (2022) e2021MS002814.
4. R. Séférian, et al. Evaluation of CNRM Earth-system model, CNRM-ESM 2-1: Role of Earth system processes in present-day and future climate. J. Adv. Model. Earth Syst. 11 (2019) 4182-4227.
5. R. Döscher, et al. The EC-Earth3 Earth system model for the Coupled Model Intercomparison Project 6, Geosci. Model. Dev. 15 (2022) 2973-3020.
6. N. P. Vuichard, et al. Accounting for carbon and nitrogen interactions in the global terrestrial ecosystem model ORCHIDEE (trunk version, rev 4999): Multi-scale evaluation of gross primary production. Geosci. Model. Dev.12 (2019) 4751-4779.
7. G. Pak, et al. Korea Institute of Ocean Science and Technology Earth System Model and Its Simulation Characteristics. Ocean Sci. 56 (2021) 18-45.
8. T. Hajima, et al. Development of the MIROC-ES2L Earth system model and evaluation of its climate–biogeochemical processes and feedbacks. Geosci. Model. Dev.13 (2019) 2197-2244.
9. T. Mauritsen, et al. Developments in the MPI-M Earth System Model version 1.2 (MPI-ESM1.2) and its response to increasing CO_2_. J. Adv. Model. Earth Syst. 11 (2019) 998-1038.
10. Ø. Seland, et al. Overview of the Norwegian Earth System Model (NorESM2) and key climate response of CMIP6 DECK, historical, and scenario simulations. Geosci. Model. Dev. 13 (2020) 6165-6200.
